# A Time-Series Analysis of the 20th Century Climate Simulations Produced for the IPCC’s Fourth Assessment Report

**DOI:** 10.1371/journal.pone.0060017

**Published:** 2013-03-28

**Authors:** Francisco Estrada, Pierre Perron, Carlos Gay-García, Benjamín Martínez-López

**Affiliations:** 1 Department of Atmospheric Sciences, Centro de Ciencias de la Atmósfera, Universidad Nacional Autónoma de México, Mexico City, Mexico; 2 Department of Environmental Economics, Institute for Environmental Studies, Vrije Universiteit Amsterdam, The Netherlands; 3 Department of Economics, Boston University, Boston, Massachusetts, United States of America; Plymouth University, United Kingdom

## Abstract

In this paper evidence of anthropogenic influence over the warming of the 20th century is presented and the debate regarding the time-series properties of global temperatures is addressed in depth. The 20th century global temperature simulations produced for the Intergovernmental Panel on Climate Change’s Fourth Assessment Report and a set of the radiative forcing series used to drive them are analyzed using modern econometric techniques. Results show that both temperatures and radiative forcing series share similar time-series properties and a common nonlinear secular movement. This long-term co-movement is characterized by the existence of time-ordered breaks in the slope of their trend functions. The evidence presented in this paper suggests that while natural forcing factors may help explain the warming of the first part of the century, anthropogenic forcing has been its main driver since the 1970’s. In terms of Article 2 of the United Nations Framework Convention on Climate Change, significant anthropogenic interference with the climate system has already occurred and the current climate models are capable of accurately simulating the response of the climate system, even if it consists in a rapid or abrupt change, to changes in external forcing factors. This paper presents a new methodological approach for conducting time-series based attribution studies.

## Introduction

For more than two decades a debate regarding the time-series properties of global and hemispheric temperatures has taken place in the climate change literature (e.g., [1]–[5]), and it has hardly been settled at the present time [6]–[10]. The underlying quest behind this discussion is the detection and attribution of climate change, both of them critical issues that have proven to be well beyond pure scientific interest, being highly relevant for example for policy- and decision-making.

This paper analyzes the time-series properties of several General Circulation Models (GCM) runs of the 20th Century Climate Experiment (20c3m) conducted for the Intergovernmental Panel on Climate Change’s (IPCC) Fourth Assessment Report (AR4) and a set of the radiative forcing series used to drive the 20c3m simulations to investigate four main issues:

1. Can the nonstationarities in global temperatures be tracked to the anthropogenic radiative forcing? Analyzing the time-series properties of climate models simulations offers the advantage of knowing the experimental design from which they were generated, therefore facilitating the detection and attribution of the nonstationarities present in temperature data.
2. Is the assumption of unit roots in global temperatures consistent with the physics of the climate system? GCM represent the state-of-the-art of climate modeling and the most advanced and complete knowledge of the physics that govern the climate system available to this date. As such, one approach for testing whether or not a unit root representation is a valid assumption for global temperatures in terms of the climate physics is to analyze the time-series properties of GCM simulations.
3. Is the unit root representation adequate for the radiative forcing series? While there has been a long debate regarding the time-series properties of global and hemispheric temperatures, radiative forcing variables have received little attention in this respect, and have usually been assumed to be integrated processes. Here we test the statistical adequacy of this assumption.
4. Are current climate models capable of reproducing important properties of observed temperature series such as structural changes and nonlinear trends? This could be considered as another characteristic to evaluate GCM performance for reproducing current climate and their ability for representing the “climate change forcing signal”.

To answer these questions, we present a new methodological approach based on recent advances in econometric methods that provides an alternative to the cointegration approach commonly used for attribution studies. As has been discussed in the literature, the latter approach could lead to incorrect inferences, such as spurious cointegration, since the data generating process of temperature series has been previously misidentified [6], [10]. In addition, the proposed methodological approach is broad enough to have wide applicability in the analysis of trending variables and their long-term relationships in climate research.

The remainder of this paper is organized as follows. The next section describes the data used and briefly discusses some advantages and limitations of the 20c3m experiment that are relevant for the purposes of our study. In the same section, the fundamental aspects of the econometric methodology are described, while a more formal discussion of the methods is offered in the online supporting information. The third section investigates the data generating processes of the simulated global temperatures and radiative forcing series using different standard unit root/stationarity tests and contrasts these results with those of a new generation unit root test that allows for a one-time break in the trend function. Attribution of climate change is then investigated using a nonlinear nonparametric co-trending test and by the analysis of the regressions residuals of global temperature simulations on radiative forcing series. The last section presents a summary of the main findings of this paper.

## Data and Methodology

### Data Description and Source

**Figure 1:**
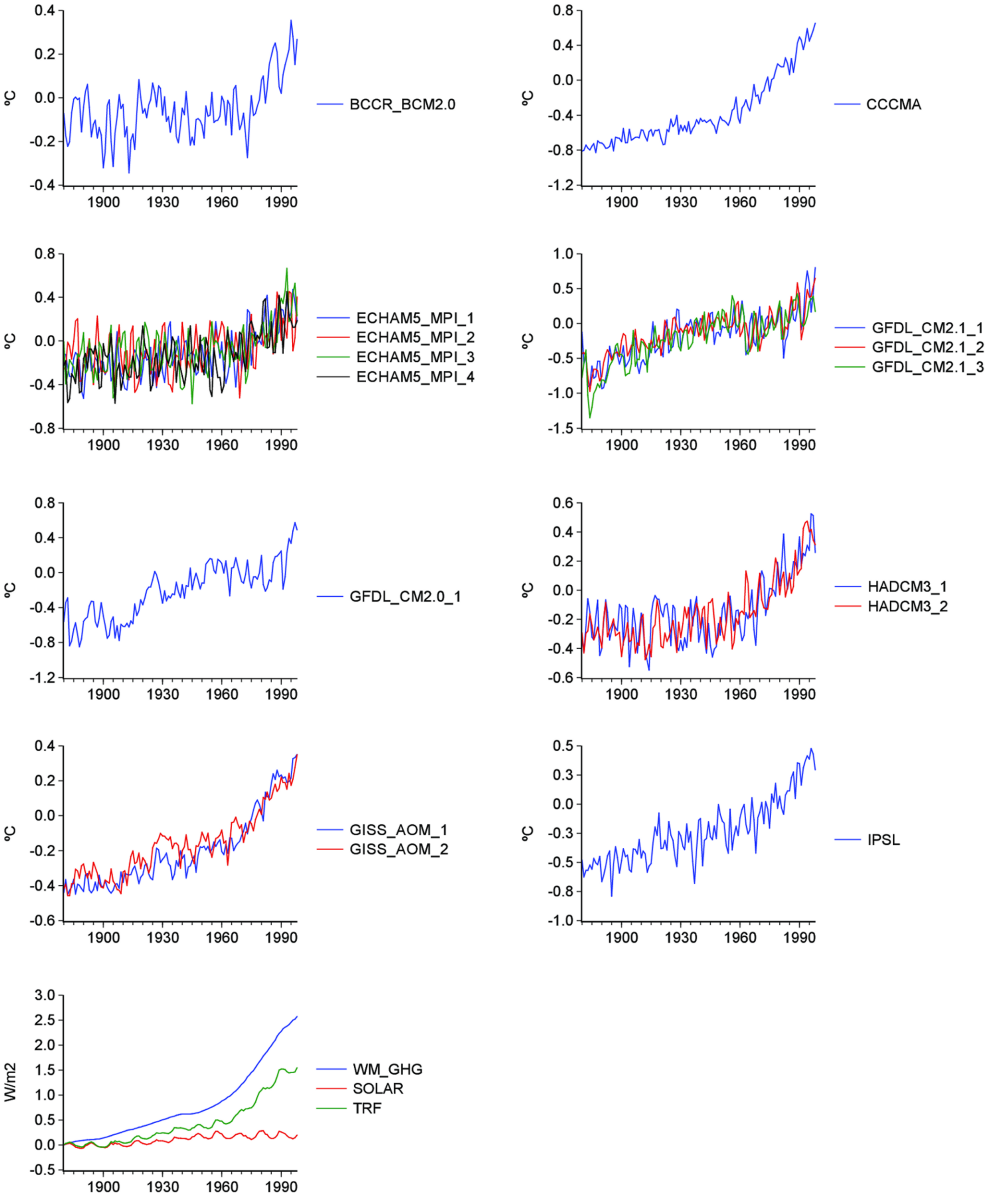
IPCC’s AR4 20 cm3 global temperature simulations and radiative forcing variables. WM_GHG includes carbon dioxide, methane, nitrous oxide and chlorofluorocarbons; SOLAR is solar forcing; TRF includes WM_GHG, solar irradiance, reflective tropospheric aerosols, indirect effect of aerosols, ozone stratospheric water vapor, land use change, snow albedo and black carbon. Climate models’ simulations are shown as anomalies with respect to their 1961–1990 mean values.

The time-series properties of 15 GCM simulations of the global 2-meter air temperature produced for the IPCC’s AR4 20c3m are analyzed. Due to the large number of realizations, using subsets of the 20c3m is a common practice when investigating particular features of this climate modeling experiment. The sample of model runs in this paper was chosen to include the most commonly used general circulation models and was influenced by data availability at the time of writing this paper. The differences in the number of runs per model depend on the modeling groups’ decisions on how many simulations they contributed to the 20c3m and on their availability. The uniformity of the results presented in the following sections suggests that similar conclusions may be expected from other 20c3m simulations. Two simulations correspond to the Bergen Climate Model version 2 of the Bjerknes Centre for Climate Research (BCCR_BCM2.0) and to the model of the Canadian Centre for Climate Modelling and Analysis (CCCMA); four to the European Centre-Hamburg model version 5 of the Max Planck Institute for Meteorology (MPI_ECHAM5); three to the Geophysical Fluid Dynamics Laboratory Climate Model version 2 of the National Ocean and Atmosphere Administration (GFDL_CM2.1) and one to the previous version of the same model (GFDL_CM2.0); two to the Hadley Centre Coupled Model version 3 (HADLEY_CM3); two to the Goddard Institute for Space Studies, Atmosphere-Ocean Model (GISS_AOM); and one to the Institute Pierre Simon Laplace climate model (IPSL). All simulations were obtained from the Royal Netherlands Meteorological Institute’s Climate Explorer (http://climexp.knmi.nl/selectfield_co2.cgi?someone@somewhere). Figure 1 plots the simulated global temperatures, and as can be seen from visual inspection the GFDL’s realizations are the noisiest with large realizations occurring in the 1880 decade. The observed global surface temperature series used in this paper corresponds to the Climate Research Unit HadCRUT3 (available at http://www.metoffice.gov.uk/hadobs/hadcrut3/).

Analyzing the time-series properties of climate models simulations offers the advantages of knowing the experimental design on which they were generated. Unlike the observed data, the 20c3m climate simulations are part of a controlled experiment for which the forcing factors that could impart secular movement to simulated global temperatures are explicitly identified. In this case, the relationship between the exogenous model inputs and endogenous model outputs is unambiguous and therefore the analysis of the radiative forcing variables can provide critical information about the warming trend of the 20th century. In particular, if the attribution of climate change is to be proven by means of currently available statistical models, both temperature and radiative forcing should share similar time-series properties, although the internal variability of climate models may modify some of their particular aspects.

In order to take advantage of the information contained in these temperature simulations, an analysis of the time-series properties of one of the sets of radiative forcing series that were used to run the 20c3m experiments is presented, and the existence of a common secular trend between temperature and forcing variables is investigated. Unfortunately, the 20c3m does not have a unique common set of radiative forcing variables and therefore simulations differ in which forcings are used (different forcing variables and sources) and on how they are incorporated into the different models [11]. The latter is particularly problematic in the case of the radiative forcing of the sulfate aerosols (direct and indirect effects) since they depend not only on the different datasets used for prescribing them [12] but also in the particular implementation of the climate model. As such, even when using the same loading patterns and time variation, the resulting radiative forcing would vary from model to model and most of these time series are not publicly available.

As a consequence, the attribution analysis presented in this paper is based on the well-mixed greenhouse radiative forcing, a variable included in all of the simulations and for which the different datasets are broadly similar. Results including other radiative forcing variables are presented to provide a sensitivity analysis to assess the robustness of our conclusions.

The radiative forcing set selected for this study is the GISS-NASA database [13] (available at http://data.giss.nasa.gov/modelforce/RadF.txt), covering the period 1880–2010 and including the following variables (in W/m^2^): well-mixed greenhouse gases (WM_GHG; carbon dioxide, methane, nitrous oxide and chlorofluorocarbons); ozone; stratospheric water vapor; solar irradiance; land use change; snow albedo; stratospheric aerosols; black carbon; reflective tropospheric aerosols; and the indirect effect of aerosols. These time series were used to construct the forcing trends in Figure 1∶1) WM_GHG, which is mostly human-induced; 2) solar forcing (SOLAR); 3) TRF, defined as the sum of all forcing variables above with the exception of stratospheric aerosols. Stratospheric aerosols can be considered stationary around a constant and therefore cannot impart the trending behavior in the level of the total radiative forcing, nor on temperature series (the Augmented Dickey-Fuller test statistic value for this series is −4.92, which is significant at the 1% level).

### Econometric Methodology

#### Unit root tests and the identification of the data generating process

Two types of nonstationary stochastic processes have been commonly proposed for modeling global temperature series: trend stationary (TS) and difference stationary (DS). These processes offer contrasting views on how the climate system works and on the importance and effects of changes in anthropogenic forcing over climate, and require different approaches for conducting time-series based attribution studies. If these processes are misidentified, a wide range of statistical models, tests and procedures can produce misleading results and inferences. A brief description of TS, DS and cointegrated processes is provided in the online supporting information (Text S1, section 1.1).

As a first step for investigating the data generating process of the simulated global temperatures and radiative forcing trend series described above, five commonly used unit root and stationarity tests are applied (Text S1, section 1.2). Nevertheless, it is important to consider that these tests can be severely affected when the trend function is subject to changes in level and/or slope. As shown in the literature, the sum of the first order autoregressive coefficients is highly biased towards unity if there is a shift in the trend function [14]. In this case, the unit root null is hardly rejected even if the series is composed of white noise disturbances around a trend. Furthermore, if the break occurs in the slope of the trend function, unit root tests are not consistent, i.e., the null hypothesis of a unit root cannot be rejected even asymptotically [15].

The existence of change points in the trend functions of temperature and radiative forcing series has been documented [16]–[20], [6], [12] and therefore standard unit root tests may not be adequate for investigating the data generating process of these variables. In consequence, we apply two new generation econometric procedures explicitly designed for addressing this problem: the Perron-Yabu structural change testing procedure [21], [22] and the Kim-Perron unit root test that allows for an unknown one-time structural break in the trend function [23]. These methodologies are briefly described in the Supporting Information (Text S1, sections 1.3 and 1.4). The main ingredient underlying the construction of the estimates and tests is the following specification of the trend function for a given series 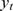:
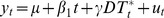
(1)where 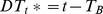 if 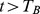 and 0 otherwise. Here 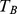 is the break date, 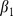 is the pre-break slope of the trend, 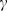
is the change in the slope at the time of the break, while 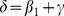 is the post-break slope. 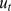 is a random process whose properties need to be investigated, i.e., stationary or integrated.

### Nonlinear Nonparametric Co-trending Test and the Attribution of Climate Change

Cointegration techniques have been commonly applied in attribution studies due to the fact that these techniques offer the possibility, under the DS assumption, of investigating the existence of a common long-term trend between temperatures and radiative forcing variables. However, unit root processes are not the only type of nonstationary processes that can show a common secular movement and cointegration analysis is only one possibility for relating the trends of nonstationary variables. Relationships between nonstationary variables can be established when linear combinations of different time series cancel out some “common features” such as trends and breaks [24].

Once we establish that global temperature simulations and radiative forcing series are better characterized as TS, we apply the nonparametric nonlinear co-trending analysis proposed by Bierens ([25]; Text S1, section 1.5) to investigate the attribution of climate change. Nonlinear co-trending is a special case of common features in which one or more linear combinations (called co-trending vectors) of nonstationary time series are stationary about a linear trend or a constant, indicating that the series share common nonlinear deterministic time trends. With r denoting the number of co-trending vectors, n series share a common nonlinear trend if one cannot reject the null hypothesis that r = n−1, while the null hypothesis that r = n can be rejected.

## Results and Discussion

### Standard Unit Root Tests

The results of applying standard unit root and stationarity tests to the global temperature models simulations and to the radiative forcing trends reveal that for all tests and series, with the possible exception of the GFDL_CM2.1 simulation 2 and the ECHAM5 simulation 4, the unit root hypothesis cannot be rejected (Table S1). Similar findings have been reported for observed global and hemispheric temperatures as well as for radiative forcing series using these tests (e.g., [4]–[6]). From these results it could be erroneously concluded that both global temperatures and radiative forcing are integrated processes and that cointegration techniques would be adequate for investigating their long-run relationships. Nevertheless, as is shown below, the finding of unit roots in global temperature and radiative forcing series is due to an incorrect specification of the trend function. Unit root tests that allow for a better representation of the trend function in these variables provide contrasting results.

### Unit Root Tests Allowing for a One-time Structural Change

As argued in the literature [6], [10], given the time-series properties of temperature series, standard unit root tests can cause to erroneously classify these series as having stochastic trends. This can also be the case for the radiative forcing series.

Visual inspection of temperature series in Figure 1 suggests the existence of structural breaks in the slope of the trend functions similar to the one in observed global temperature series discussed in previous publications (e.g., [6], [16]–[17]). The existence of changes in the rates of growth of the various greenhouse gases is frequently discussed in the climate policy and mitigation contexts (e.g., [19], [20]) and is also clearly suggested by Figure 1. Therefore, it is important to assess whether the results from standard unit root tests are affected by the presence of structural changes. However, this is a circular problem given that most of the tests for structural breaks require to correctly identify if the data generating process is stationary or integrated. Depending on this outcome, the limit distribution of these tests are different and, if the process is misidentified, the tests will have poor properties. The Perron-Yabu procedure offers a way to break this circular problem allowing to test for structural changes in level and/or slope whether the noise component is stationary or integrated [21], [22].

The results of this procedure are presented in Table 1 column 3. The test statistic values for all temperature simulations are significant at the 5% level, with the exception of GFDL_CM2.1_3 which is significant at the 10% level and of GFDL_CM2.1_2 which is not significant at any conventional levels (not reported in Table 1). In the case of the forcing variables TRF and WM_GHG the test statistic values are significant at the 1% levels, while for SOLAR it is at the 10% level, indicating in all cases the presence of structural changes in their rates of growth.

**Table 1:** Tests for a unit root with a one-time break in the trend function.

Series	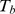	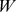	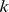	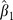	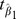	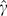	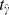	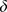	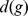	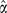	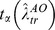
OBSERVED	1977	3.59^a^	0	0.0035	**10.84**	0.0142	**7.85**	0.0177	0.00%	0.50	−5.73^a^
ECHAM5_1	1968	8.04^a^	1	0.0011	**2.63**	0.0135	**8.29**	0.0146	−17.84%	0.04	−9.20^a^
ECHAM5_2	1978	4.55^a^	2	0.0015	**3.41**	0.0167	**6.22**	0.0182	2.61%	0.16	−9.77^a^
ECHAM5_3	1973	8.27^a^	1	0.0010	**2.24**	0.0161	**7.45**	0.0171	−3.59%	−0.07	−4.86^a^
ECHAM5_4	1961	3.76^a^	2	0.0013	**2.70**	0.0100	**5.45**	0.0114	−35.78%	0.25	−5.81^a^
BCCR	1974	2.32^b^	0	0.0004	1.57	0.0136	**7.82**	0.0140	−20.93%	0.50	−5.88^a^
CCCMA	1961	5.80^a^	0	0.0042	**19.07**	0.0230	**32.55**	0.0273	53.82%	0.27	−8.35^a^
GFDL_CM2.1_1	1888	1.95^b^	2	−0.0086	−**2.71**	0.0166	**4.69**	0.0079	−55.68%	0.46	−4.51^a^
GFDL_CM2.1_3	1885	1.72^c^	2	−0.0062	−1.47	0.0144	**3.19**	0.0083	−53.18%	0.59	−7.37^a^
GFDL_CM2.0_1	1889	2.53^b^	0	−0.0152	−**6.45**	0.0231	**8.87**	0.0079	−55.22%	0.65	−4.44^a^
HADLEY_CM3_1	1963	9.59^a^	2	0.0007	1.76	0.0161	**10.33**	0.0167	−5.80%	0.30	−5.15^a^
HADLEY_CM3_2	1958	6.59^a^	0	0.0010	**2.84**	0.0127	**10.18**	0.0137	−22.86%	0.36	−7.47^a^
GISS_AOM_1	1966	13.67^a^	0	0.0030	**23.07**	0.0124	**20.70**	0.0154	−13.20%	0.35	−7.37^a^
GISS_AOM_2	1973	5.93^a^	0	0.0035	**22.35**	0.0107	**10.99**	0.0142	−19.84%	0.55	−5.56^a^
IPSL	1969	10.99^a^	0	0.0037	**10.69**	0.0163	**9.25**	0.0200	12.46%	0.17	−8.80^a^
TRF	1960	5.63^a^	2	0.0064	**20.82**	0.0221	**28.98**	0.0285	–	0.84	−4.24^b^
WM_GHG	1960	62.79^a^	7	0.0105	**64.95**	0.0351	**87.76**	0.0456	–	0.90	−3.97^b^
SOLAR	1959	1.80^c^	2	0.0031	**16.33**	−0.0032	−**6.793**	−0.0001	–	0.58	−8.82^a^

The regression model for the unit root tests is defined in equations (4) and (6) in the Supporting Information. The symbols are defined as follows: *T_b_* is the estimated time of the break; 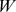 is the Perron-Yabu Exp-Wald statistic with 5% trimming; *k* is the number of lagged differences added to correct for serial autocorrelation; 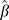, 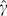 are the regression coefficients of the slope of the trend function and 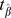, 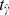 the corresponding t-statistic values. Bold numbers denote statistical significance at 5% levels. 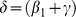 is the post-break slope and 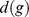 is the percent difference with respect to the observed global temperature. 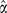 is the sum of the first order autoregressive coefficients and 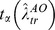 is the Kim-Perron unit root test statistic.

a, b, c denotes statistical significance at the 1%, 5% and 10%, respectively (for 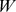, for critical values taken from [21], Table 2.b; Kim-Perron unit root test critical values taken from [39], Table 1). Results for Observed taken from [6].

Consequently, unit root tests that allow for possible structural changes are required for investigating the type of data generating process that best describes temperature and forcing series. For this task, the Kim-Perron unit root test was applied and, as discussed below, once a break in the trend function is allowed the results of standard unit root tests are completely reversed.

The results in Table 1 are quite striking and uniform across all series clearly rejecting the null hypothesis of a unit root at the 1% significance level, for all of the model simulations (see Text S1 section 1.4.1 for a robustness analysis of the unit root test results). In the case of the GFDL_CM2.1_2 simulations the Perron-Yabu test does not reject the null of no break. However, the ADF test with no break rejects the null hypothesis of a unit root for this series (Table S1). Hence, we can conclude that it is TS and no further analysis is needed. As expected from TS series, the estimates of the sum of the autoregressive coefficients of the simulated temperature series are now quite far from unity, ranging from -0.07 (ECHAM5_3) to 0.65 (GFDL_CM2.0_1), with a mean value of 0.34. As in the case of observed global temperature reported previously in the literature, assuming a unit root would have erroneously attributed too much persistence to temperature variability, a fact not supported by the data [6].

While there has been a debate regarding the time-series properties of global and hemispheric temperatures, radiative forcing variables have received little attention in this respect and have usually been assumed to be integrated processes when conducting attribution studies based on observed records and time series analysis [4]–[5]. The two main arguments for justifying this assumption are: 1) the results of standard unit root tests, which as discussed above are not adequate for this task given the presence of structural breaks, and; 2) the long residence time of greenhouse emissions in the atmosphere produces an accumulation process. However, it should be noticed that cumulative processes are not necessarily unit root processes, any type of trending process would produce the same effect.

The last column of Table 1 reveals that when allowing for a better representation of the trend function, the conclusions that can be drawn are markedly different from what has been reported previously in the literature. The null of a unit root is strongly rejected in favor of trend stationary processes with a one-time permanent break in the rate of growth of the forcing variables.

Although radiative forcing variables are more persistent than temperatures, the sum of the autoregressive coefficients is far from unity. Shocks in concentrations and radiative forcing do dissipate as opposed to the case of a unit root in which the persistence of shocks is infinite. This finding has important implications for the attribution of climate change since it shows that there are no differences in the order of integration of these variables and that all of them can be better described as trend stationary processes with a change in their rates of growth.

The dates of the break in the slope of the trend of the simulated temperatures vary from 1885 to 1978 (Table 1, column 2). This wide range is mainly due to the GFDL simulations which show large realizations (possible outliers) around the 1880’s decade that may affect the estimation of the break date. If these simulations are excluded, the average break date is 1968 which is close to those that have been reported in the literature [16]–[19], [26]–[27]. The break dates in the slope of the radiative forcing trends are estimated around 1960, previous to those of observed and simulated global temperatures (Table 1, column 2).

**Figure 2:**
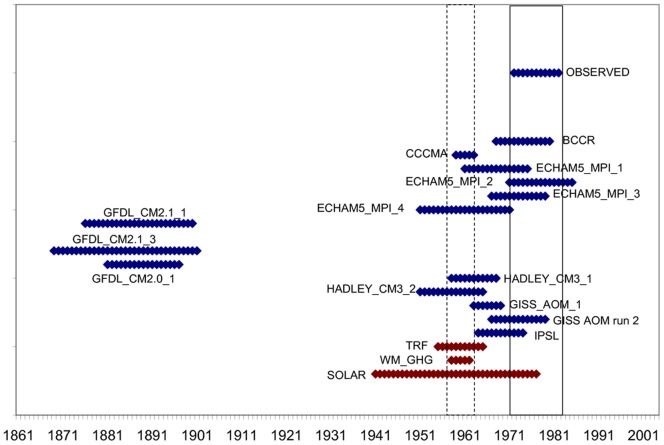
Confidence intervals for the break dates in Table 1. Solid and dashed lines indicate the 95% confidence interval for the break dates for observed global temperatures and WM_GHG, respectively.

Confidence intervals for the break dates in Table 1 were constructed using the Perron-Zhu procedure [28]. Figure 2 shows that for almost half of the model simulations, the estimated break date is not statistically different from that of the observed series. Excluding the GFDL models, although the confidence intervals do not necessarily overlap with the observed one, they are separated by only a few years and most of them cannot be considered statistically different from each other. Furthermore, with the exception of GFDL_CM2.1, all of the models for which more than one run was considered (ECHAM5, HADC3M, GISS_AOM) provide similar estimates of the break date from run to run.

The break dates of the radiative forcing trend variables are neither statistically different from each other nor from about half of the temperature simulations. However, the break date of observed global temperatures is statistically different from those of TRF and WM_GHG. The apparent delay in the response of the climate system could be related to a change in the Atlantic Multidecadal Oscillation (AMO) to its negative phase around the early 1960s, possibly obscuring the global increase in temperatures due to anthropogenic forcing [29]–[30]. One possible factor contributing to the differences in the break dates between the observed and simulated series could be associated to the fact that the 20c3m simulations are not constrained to reproduce observed variability. Therefore, natural variability and the models’ internal variability do not have to match and neither do the occurrence of changes in the phase of AMO [31]–[32]. Furthermore, current climate models tend to underestimate inter-annual low-frequency natural climate variability, producing fewer deviations (and of shorter duration) that could mask the warming trend [12].

The fact that runs from different models and models with multiple runs that have similar or identical forcing but different initial conditions give broadly similar estimates of the break date provides further evidence of its exogenous nature: this common feature of model simulations cannot be interpreted as part of internal variability, but as a result of the changes in radiative forcing.

**Figure 3:**
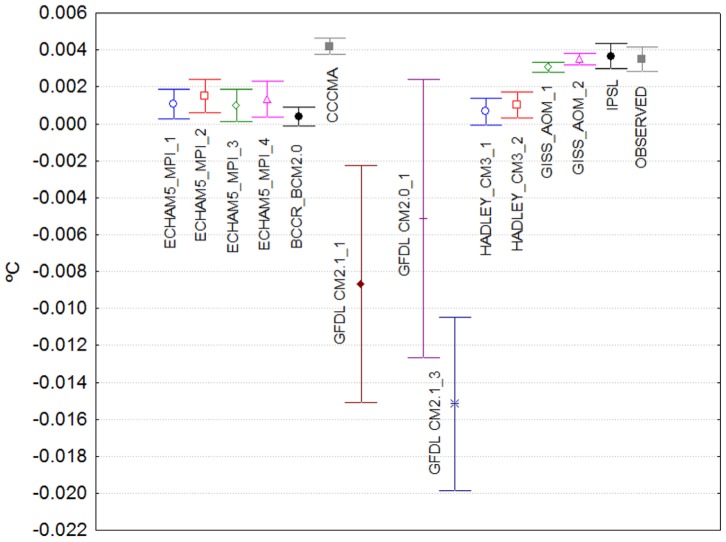
Point estimates and 95% confidence intervals of the pre-break slope coefficients (°C/yr) in Table 1.

Figures 3A and 3B show the point estimates and the corresponding 95% confidence intervals of the coefficients of the pre-break slopes and of their changes after the break, respectively. For most of the simulations, a positive and statistically significant pre-break trend is present, nevertheless the coefficients are not statistically different from that of the observed temperature series only for IPSL, GISS_AOM, CCCMA models (Figure 3). When comparing the magnitude of the pre-break slope coefficients of the model simulations with that of the observed one, even if the GFDL models are excluded (for this model the range of the estimates of the pre-break slope coefficient vary from −534.29% to 20% in comparison with the observed estimate), the differences are quite large and the range of values span from −88.57% to 20%. Most of the models underestimate the first warming trend of the 20th century, possibly due to large realizations of observed natural variability [33].

**Figure 4:**
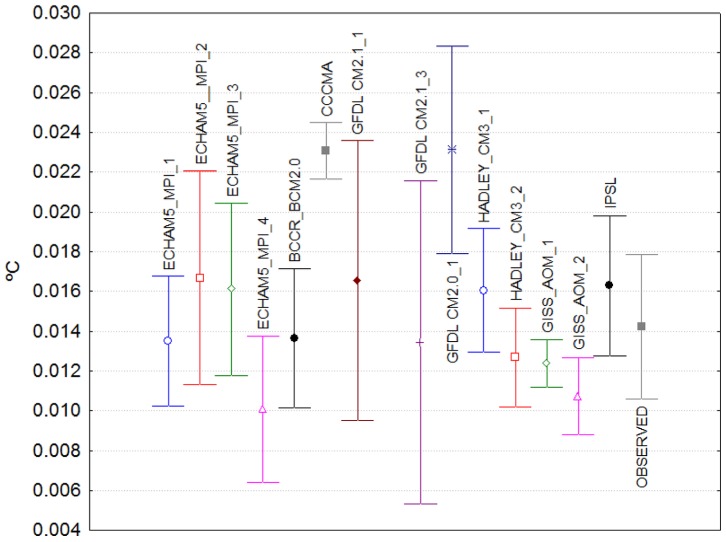
Point estimates and 95% confidence intervals of the post-break change coefficients (°C/yr) in Table 1.

In contrast, the changes in the slope coefficients induced by the structural change are not statistically different from each other for all the simulated and observed temperature series, with the exception of CCCMA (Figure 4). The similitude in these parameter values provides evidence to support the fact that climate models can accurately simulate the response of the climate system to changes in external forcing factors, even if rapid or abrupt, and therefore gives more confidence in their ability to produce credible climate change scenarios at least at the global scale. Note however that, as has been discussed in the literature, this high level of agreement between models occurs despite their large differences in key factors such as climate sensitivity and climate forcing (e.g., [34]–[35]).

Finally, when comparing the post-break slope value (pre-break plus change in slope at the break) to that of the observed global temperature, it becomes apparent that, at least in this sample of models and simulations, climate models included in the IPCC’s AR4 tend to underestimate the warming trend that was observed in the second part of the 20th century. As depicted by columns 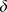 and 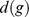 in Table 1, twelve of the models simulations underestimate the observed trend of the last part of the century (some of them severely, up to 65%). The remaining simulations show from slight overestimations (ECHAM5_2 and IPSL) to large overestimations (CCCMA, about 50%).

### Attribution of the 20th Century Warming Trend

**Figure 5:**
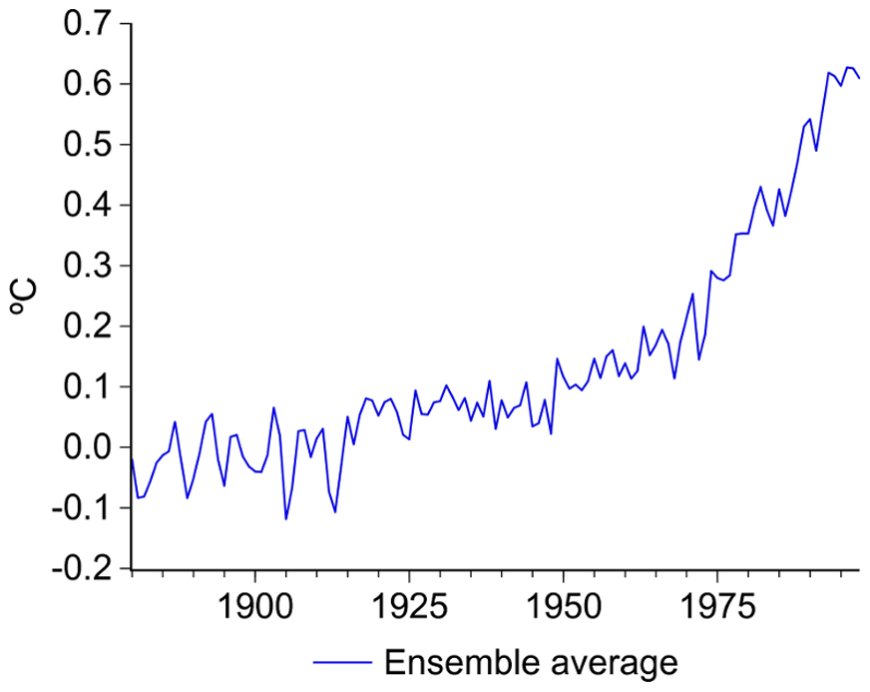
Average of models runs (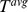).

A nonparametric nonlinear co-trending test [25] is applied to investigate if the radiative forcing trends, in particular WM_GHG, and the average of the 20c3m simulations (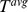, depicted in Figure 5) share a common nonlinear trend. An analysis of the residuals of ordinary least squares regressions is also presented to further illustrate the existence of common secular trends. The GFDL’s simulations were excluded because of their poor performance in reproducing the observed global trend. However, the results presented below are robust to the inclusion of the GFDL simulations.

The co-trending test results provide strong evidence for the attribution of climate change to the anthropogenic forcing represented by WM_GHG, an input common to all of the 20c3m simulations, and shows that the existence of a common nonlinear trend is robust to the inclusion of other forcing factors. The empirical evidence obtained by this test can be summarized as follows (see Table S2):

1. There is a unique co-trending vector (r = 1) between 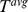 and WM_GHG, indicating that these variables share a common nonlinear trend.
2. The existence of a unique co-trending vector is robust to the inclusion of all the other forcing factors in TRF.
3. TRF, WM_GHG and 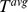 share the same nonlinear trend (two co-trending vectors, r = 2).
4. SOLAR and 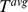 show a distinct long-run secular movement, suggesting that the observed warming can hardly be approximated by the main natural factor (r = 0).
5. There is a unique co-trending vector (r = 1) between 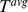 and the observed global temperature series.

These results not only support the findings in the previous subsections regarding that temperature and radiative forcing variables are stationary around a common nonlinear trend, but provide strong evidence of attribution of the warming of the 20th century to anthropogenic activities. Results 1, 2 and 3 suggest that, although other forcing factors have had an important effect modulating the net forcing, the nonlinear trend defining the secular movement of TRF and global temperatures during the past century is largely defined by that of WM_GHG. Result 5 shows that, in spite of the ensemble’s members differences reported above, the observed and the average of the simulated global temperatures share the same nonlinear trend, further confirming the ability of current GCM to reproduce the 20th century warming trend.

Given that both radiative forcing and global temperature series have been shown to be TS processes, their long-term relationship can also be investigated using simple OLS regressions involving global temperature and radiative forcing series as the dependent and independent variables, respectively, and analyzing the associated residuals.

**Figure 6:**
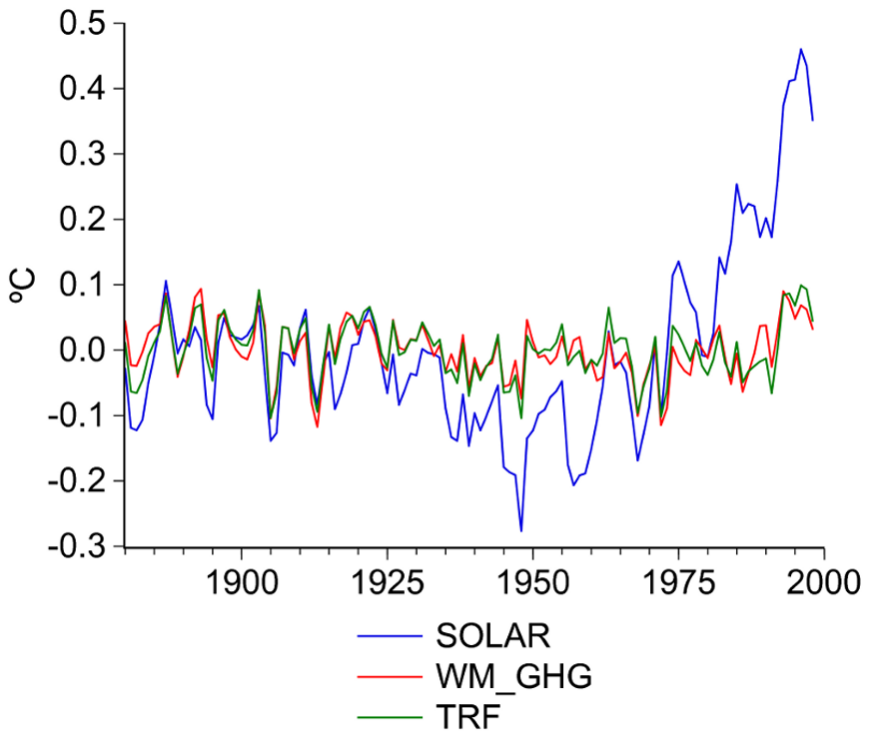
Residuals of the regressions of 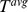 on SOLAR, TRF and WM_GHG. 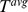
 is the average of models runs; WM_GHG includes carbon dioxide, methane, nitrous oxide and chlorofluorocarbons; SOLAR is solar forcing; TRF includes WM_GHG, solar irradiance, reflective tropospheric aerosols, indirect effect of aerosols, ozone stratospheric water vapor, land use change, snow albedo and black carbon.

The residuals from the regression of 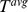
on SOLAR reveal that the trend of this variable could only account for part of the warming in the first half of the 20th century (Figure 6). The large positive trend in the residuals since the 1950s confirms that these variables follow different secular trends. However, when using TRF or WM_GHG as the explanatory variable the residuals of the regression are stationary, indicating that these series can indeed reproduce the nonlinear warming trend of the 20th century. As such, visual inspection of the residuals strongly suggest that the main source of the secular movement in both TRF and global temperatures is WM_GHG, although other internal and external forcing factors have modulated them.

The ADF test [36]–[37] with no deterministic terms (Table S3) confirms that both the residuals from the regressions using TRF and WM_GHG can be considered as stationary variations around the zero line (the test statistics are about 2.5 times the 1% critical value), while the residuals obtained from a regression using SOLAR are clearly nonstationary (the test statistic is not significant at any conventional level).

Overall, the results are in strong agreement with previous attribution studies based on GCM simulations under different combinations of external forcing factors, indicating that the warming of the 20th century cannot be reproduced without the inclusion of the main anthropogenic forcing factors [12], [26]–[27], [30], [38].

### Conclusions

This paper presents a new approach for investigating the attribution of climate change based on state-of-the-art econometric techniques that are appropriate for the time-series properties of global temperature and radiative forcing. The results reveal sound statistical evidence underlying the large anthropogenic contribution to the warming of the 20th century. It is shown that WM_GHG, TRF and 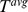 share a unique common nonlinear trend which is also shown to match the warming trend in observed global temperatures. In contrast, the nonlinear trend describing the secular movement of solar forcing is statistically distinct from that of the observed and simulated temperatures, being particularly unable to explain their evolution during the second part of the century.

By means of new generation unit root and structural change testing procedures, strong evidence is presented suggesting that both global temperatures and radiative forcing series have been misidentified in previous studies as being unit root processes. All these series share similar time-series properties and can be better characterized as stationary processes around nonlinear deterministic trends with time-ordered breaks that are spaced in a way consistent with what could be expected from climate physics. Given the experimental design of the 20c3m and the similitude between different models and runs, this finding provides an unambiguous causal explanation for the increase in the rate of warming during the second part of the 20th century.

The results offer additional evidence regarding the capacity of current climate models to accurately simulate the response of the climate system to changes in external forcing factors, even if rapid or abrupt. This finding contributes to increase confidence in the ability of these models to produce credible climate change scenarios at least for such large spatial scales.

## Supporting Information

Table S1 **Standard unit root tests applied to global temperature simulations and radiative forcing series.**
(PDF)

Table S2
**Nonparametric nonlinear co-trending test for TRF, SOLAR, WM_GHG, 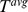 and 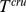.**
(PDF)

Table S3 **ADF test on the residuals of the regressions of the ensemble average of global temperature simulations on: 1) TRF; 2) WM_GHG and; 3) SOLAR.**
(PDF)

Text S1 **Supplementary methods and results.**
(PDF)
