# Supplementary material for: A Time-Series Analysis of the 20th Century Climate Simulations Produced for the IPCC’s Fourth Assessment Report
Source: PLoS One. 2013 Mar 28;8(3):e60017. doi: 10.1371/journal.pone.0060017 (PMC3610752; doi:10.1371/journal.pone.0060017)
Supplement: Table S1 — Standard unit root tests applied to global temperature simulations and radiative forcing series. (PDF) [file pone.0060017.s001.pdf]

Table S1. Standard unit root tests applied to global temperature simulations and radiative forcing series.The entries show the test statistic value of the corresponding unit root/stationarity test. The lag length is given in parentheses. The model specification includes a constant and a linear trend. Figures in bold indicate that the test statistic is significant at the 5% level.

Series	ADF	DF-GLS	KPSS	ERS-PO	Ng-Perron		
CCCMA	1.2286	0.3160	**0.3556**	191.8251	0.7635	(MZa)	(3)
	(5)	(4)		(5)	0.3572	(MZt)	
					0.4679	(MSB)	
					59.109	(MPT)	
ECHAM5 MPI 1	-1.5503	-1.1852	**0.3362**	32.0253	-2.6775	(MZa)	(7)
	(7)	(7)		(7)	-1.0730	(MZt)	
					0.4007	(MSB)	
					31.285	(MPT)	
ECHAM5MPI 2	-0.9211	-1.1370	**0.2726**	29.1359	-0.1321	(MZa)	(7)
	(11)	(11)		(11)	-0.2265	(MZt)	
					1.7139	(MSB)	
					538.580	(MPT)	
ECHAM5 MPI 3	-1.2011	-1.2635	**0.2852**	35.6076	-2.1167	(MZa)	(11)
	(9)	(9)		(9)	-0.9619	(MZt)	
					0.4544	(MSB)	
					39.445	(MPT)	
ECHAM5 MPI 4	**-4.7074**	-2.2816	**0.2279**	**4.1871**	-1.1700	(MZa)	(12)
	(2)	(5)		(2)	-0.5147	(MZt)	
					0.4399	(MSB)	
					43.147	(MPT)	
HADLEY CM3 1	-2.7749	-2.8283	**0.2936**	6.1165	-3.12956	(MZa)	(10)
	(2)	(2)		(2)	-1.16178	(MZt)	
					0.37123	(MSB)	
					27.0944	(MPT)	
HADLEY CM3 2	-0.3803	-0.4268	**0.3192**	52.0889	-1.6343	(MZa)	(7)
	(7)	(7)		(7)	-0.6939	(MZt)	
					0.4246	(MSB)	
					38.7708	(MPT)	
GFDL CM2.0 1	-2.1086	-1.8418	**0.1570**	12.7112	-7.6997	(MZa)	(3)
	(3)	(3)		(3)	-1.8047	(MZt)	
					0.2344	(MSB)	
					12.2293	(MPT)	
GFDL CM21 1	-2.4141	-2.1679	0.1147	12.1291	-4.5621	(MZa)	(7)
	(4)	(4)		(4)	-1.3217	(MZt)	
					0.2897	(MSB)	
					18.6679	(MPT)	
GFDL CM21 2	**-3.8855**	**-3.7399**	0.0748	3.9746	-10.511	(MZa)	(7)
	**(2)**	**(2)**		(2)	-2.1926	(MZt)	
					0.2086	(MSB)	
					9.1576	(MPT)	
GFDL CM21 3	-3.4215	-2.6597	0.1014	6.7365	-10.462	(MZa)	(4)
	(3)	(3)		(3)	-2.2831	(MZt)	
					0.2182	(MSB)	
					8.7303	(MPT)	
GISS AOM 1	-0.4115	-0.6654	**0.3031**	73.7630	-0.7532	(MZa)	(5)

	(5)	(3)		(5)	-0.3639	(MZt)	
					0.4831	(MSB)	
					51.866	(MPT)	
GISS AOM 2	**-3.9531**	-2.5734	**0.2048**	5.7746	-2.7044	(MZa)	(6)
	**(0)**	(1)		(0)	-0.80368	(MZt)	
					0.29718	(MSB)	
					23.615	(MPT)	
IPSL	-0.7816	-0.0531	**0.3023**	128.1323	-0.0015	(MZa)	(8)
	(8)	(8)		(8)	-0.0009	(MZt)	
					0.6337	(MSB)	
					87.555	(MPT)	
TRF	-2.7258	-2.2095	**0.3203**	**0.0966**	-6.5087	(MZa)	(9)
	(5)	(11)		(12)	-1.7097	(MZt)	
					0.2627	(MSB)	
					14.040	(MPT)	
WM_GHG	-2.7440	-2.8414	**0.3387**	**4.6058**	**-198.70**	(MZa)	(11)
	(7)	(7)		(8)	**-9.9324**	(MZt)	
					**0.0500**	(MSB)	
					**0.5569**	(MPT)	
SOLAR	0.6108	-0.8866	**0.2674**	125.9262	-0.1105	(MZa)	(8)
	(8)	(9)		(8)	-0.0721	(MZt)	
					0.6522	(MSB)	
					90.547	(MPT)	
