# Supplementary material for: A Time-Series Analysis of the 20th Century Climate Simulations Produced for the IPCC’s Fourth Assessment Report
Source: PLoS One. 2013 Mar 28;8(3):e60017. doi: 10.1371/journal.pone.0060017 (PMC3610752; doi:10.1371/journal.pone.0060017)
Supplement: Table S2 — Nonparametric nonlinear co-trending test for TRF, SOLAR, WM_GHG, and . (PDF) [file pone.0060017.s002.pdf]

Table S2. Nonparametric nonlinear co-trending test for TRF, SOLAR, WM_GHG, T^{avg} and T^{cru}. Bold figures denote statistical significance at the 10% level.

r	WM_GHG, T^{avg}	TRF, T^{avg}	TRF,WM_GHG, T^{avg}	SOLAR, T^{avg}	T^{cru}, T^{avg}
1	0.05789	0.08033	0.05578	**0.14461**	0.05195
2	**0.31774**	**0.30835**	0.09681	**0.30793**	**0.31276**
3	--	--	**0.32841**	--	--
