# Supplementary material for: A Time-Series Analysis of the 20th Century Climate Simulations Produced for the IPCC’s Fourth Assessment Report
Source: PLoS One. 2013 Mar 28;8(3):e60017. doi: 10.1371/journal.pone.0060017 (PMC3610752; doi:10.1371/journal.pone.0060017)
Supplement: Table S3 — ADF test on the residuals of the regressions of the ensemble average of global temperature simulations on: 1) TRF; 2) WM_GHG and; 3) SOLAR. (PDF) [file pone.0060017.s003.pdf]

Table S3. ADF test on the residuals of the regressions of the ensemble average of global temperature simulations on: 1) TRF; 2) WM_GHG and; 3) SOLAR.

The model specification includes no deterministic term. The lag length used is given in parentheses and was chosen using the BIC. Figures in bold indicate that the statistic is significant at the 1% level.

| Forcing trend | ADF test statistic |
|---|---|
| TRF | **-6.58** |
| | (0) |
| WM_GHG | **-6.83** |
| | (0) |
| SOLAR | -1.53 |
| | (0) |
