# Supplementary material for: A Time-Series Analysis of the 20th Century Climate Simulations Produced for the IPCC’s Fourth Assessment Report
Source: PLoS One. 2013 Mar 28;8(3):e60017. doi: 10.1371/journal.pone.0060017 (PMC3610752; doi:10.1371/journal.pone.0060017)
Supplement: Text S1 — Supplementary methods and results. (PDF) [file pone.0060017.s004.pdf]

Text S1

A time-series analysis of the 20th century climate simulations produced for the IPCC's AR4

F. Estrada, P. Perron, C. Gay-García, B. Martínez-López

1.1. DS, cointegrated processes and TS

If a series is stationary around an appropriately defined trend it is said to be integrated of order zero or I(0), if the deviations from the trend have to be differenced once to achieve stationarity it is I(1), or I(2) if it has to be differenced twice. An example of an I(1) process is a first order autoregressive process, in which the coefficient of the autoregressive term is equal to one, e.g.,

$$y_t = y_{t-1} + e_t \quad ....(1)$$

or

$$\Delta y_t = e_t$$

where $\Delta = (1-L)$ is the difference operator, $e_t \sim i.i.d(0, \sigma^2)$ is a white noise process, which could be extended to an ARMA process satisfying the stationarity and invertibility conditions. This model, also known as random walk, has a stochastic trend, as can be shown by solving the difference equation (1):

$$y_t = y_0 + \sum_{i=0}^{t-1} e_{t-i}$$

where y_0 is the initial condition and $\sum_{i=0}^{t-1} e_{t-i} = v_t$ has a stochastic trend, produced by the sum of the stationary error term [1]. The mean of the process is constant and its variance increases with time $Var(y_t) = E(v_t^2) = t\sigma_e^2$ and diverges as $t \to \infty$ [2]. A generalization of equation (1) is a random walk with a drift (a constant term):

$y_t = \beta + y_{t-1} + e_t \ldots.(2)$

or

$\Delta y_t = \beta + e_t$

The solution of this difference equation is

$$y_t = y_0 + \beta t + \sum_{i=0}^{t-1} e_{t-i}$$

where y_0 is the initial condition, βt is a deterministic trend and $\sum_{i=0}^{t-1} e_{t-i} = v_t$ has a stochastic trend. The variance of this process $Var(y_t) = E(v_t^2) = t\sigma_e^2$ is time dependent as in the case of a simple random walk, but the mean $E(y_t) = y_0 + \beta t$ is no longer constant.

Two of the most important features of this type of process are that it is not mean reverting and that it has infinite memory; shocks do not fade [1]-[2]. The sum of these random shocks determines the secular movement of the series. That is, all shocks have permanent effects on the long-run path of temperature. Past and present shocks are as important for determining the current trend: any shock that occurred even in the

distant past is as important as present variations. The long-term forecast is always influenced by historical events, and temperature predictability is limited, even if forcing factors are held constant [3]-[4]. It is worth noting that this is not consistent with the physical fact of climate being a highly dissipative system [5].

Two integrated variables are said to be cointegrated if there exists a linear combination of them that produces stationary residuals [6]. Cointegration implies that there is a long-run equilibrium relationship between two or more variables because they share the same stochastic trend [7]. To illustrate this concept consider the following integrated processes:

$$x_t = \mu_{xt} + \varepsilon_{xt}$$

$$z_t = \mu_{zt} + \varepsilon_{zt}$$

where μ_{xt}, μ_{zt} are unit root processes wich contain a stochastic trend and ε_{xt}, ε_{zt} are stationary noise processes. For these two variables to be cointegrated there must exist a linear combination $\alpha_1 x_t + \alpha_2 z_t$ (α_1, α_2 are different from zero) such that $\mu_{xt} - \frac{\alpha_2}{\alpha_1}\mu_{zt}$ is stationary, indicating that the stochastic trends μ_{xt}, μ_{zt} are identical up to a scalar. Consequently, x_t and z_t share a common secular movement and a regression between these two variables will produce stationary residuals.

In the application of cointegration techniques to climate variables this common stochastic trend has been interpreted as the fingerprint of anthropogenic activities in global and hemispheric temperatures. It should be noted however that a requirement for this concept is *the existence of stochastic trends*. If the variables are for example

I(0) and I(1) the cointegration representation does not exist and spurious cointegration is likely to occur (e.g., [8]-[10]).

On the other hand, a trend stationary process consists of a deterministic component plus a stochastic process which can range from a simple white noise to a variety of different types of autoregressive and moving average structures such as AR, MA, ARMA. A simple example of this class of process is an AR(1) equation of the form:

$$\begin{aligned} x_t &= \alpha + \tau_t + v_t \\ v_t &= \phi v_{t-1} + e_t \end{aligned} \qquad (3)$$

where ϕ is a constant satisfying $|\phi| \prec 1$, $e_t \sim i.i.d\left(0, \sigma^2\right)$ is a white noise process which could also be extended to an ARMA process satisfying the stationarity and invertibility conditions, τ_t can be any deterministic function of time producing a variety of linear and nonlinear trends and α is the intercept of the trend function. The deterministic component of this process dominates its long run behavior: variations are transitory and do not change the long run path of the series [11]. These processes are mean reverting around a trend function of the form $E(x_t) = \alpha + \tau_t$. Local scale monthly temperatures in low and middle latitudes frequently provide examples of trend stationary processes, for which standard unit root test are able to strongly reject the null hypothesis of the presence of stochastic trends [e.g., 4].

It has been shown that when considering the problem of investigating the data generating process, care must be exercised when the trend function is subject to changes in level and/or slope [12]. The class of models considered in [12] are special cases for which the trend function changes only once in the sample. In this case, the

usual strategy is to treat such changes as exogenous and they are not explicitly modeled via a parametric stochastic structure. Under this parameterization, there are only some shocks that can change the long-term behavior of the time series, as opposed to the case of a unit root where all shocks produce long-term changes. In the climate context, long-term changes are not frequent in the scale of the sample under analysis and are produced by important changes in key external forcing factors such as Earth orbit changes, solar irradiance, and greenhouse gases concentrations [3].

In general, the trend parameters and their structural changes are not assumed to be deterministic [12]-[13]. In order to illustrate the class of model that applies in such cases, consider the following framework [13]:

$$y_t = \mu_t + \beta_t t + Z_t$$

$$A(L)Z_t = B(L)e_t \,;\; e_t \sim i.i.d.(0,\sigma_e^2)$$

$$\beta_t = \beta_{t-1} + u_t$$

$$\mu_t = \mu_{t-1} + v_t$$

where *A(L)* and *B(L)* are polynomials in the lag operator *L* defined as $LX_t = X_{t-1}$. The intercept and slope of the trend function are time varying stochastic processes. The noise components u_t and v_t are modeled as mixtures of normal distributions where the realizations from each of these variables are drawn from one of two normal distributions, one with high and the other with small or zero variance. These mixtures of normal distributions for the error terms u_t and v_t can be described as:

$$u_t = \lambda_t \gamma_{1t} + (1-\lambda_t)\gamma_{2t}$$

$$v_t = \kappa_t \delta_{1t} + (1 - \kappa_t)\delta_{2t}$$

where $\gamma_{it} \sim i.i.d.N(0,\sigma_{\gamma i}^2)$, $\delta_{it} \sim i.i.d.N(0,\sigma_{\delta i}^2)$ while λ_t and κ_t are Bernoulli variables that take value one with probability α_λ and α_κ and zero with probability (1-α_λ) and (1-α_κ), respectively. One can then obtain a model with infrequent changes in the slope and intercept parameters when α_λ and α_κ are close to one and $\sigma_{\gamma 1}^2$ and $\sigma_{\delta 1}^2$ are zero. If $\sigma_{\gamma 2}^2 > 0$ there will be occasional changes in the slope, and correspondingly if $\sigma_{\delta 2}^2 > 0$ there will be infrequent changes in the intercept. As mentioned above, in the case of climate change these breaks in the trend function are driven by changes in key external forcing factors. When only one break occurs, it becomes difficult to model the change with a stochastic structure. Hence, the common approach in the literature has been to consider the change as being 'exogenous'. We shall adopt this approach in our various analyses.

The occurrence of secular co-movement is not restricted to integrated variables and cointegration is only a particular case of a broader class of processes that share common time-series features. Trend stationary processes such as those described above can also exhibit a common secular movement represented by a variety of linear and nonlinear deterministic trends that may include characteristic features such as breaks in the trend function. The concept of nonlinear co-trending applied in this paper is described in section 1.5 of the present supplementary text.

1.2. Standard unit root tests and the lag length and bandwidth selection

In this paper five of the must commonly used unit root/stationarity tests were applied to the simulated global temperature and radiative forcing series [14]-[18]. A

description of these unit root tests and a discussion of their similarities and differences is available in the literature (e.g., [1]). For the ADF and DF-GLS tests the lag length was selected using the Akaike Information Criterion. For the KPSS test, the Bartlett kernel is used with the bandwidth selected using the Newey-West method [19] which automatically selects, by means of a nonparametric approach, the number of autocovariances to use when computing a hetersokedasticity and autocorrelation consistent covariance matrix. For the ERS-PO, the autoregressive spectral density estimator is used with the lag length selected using the Akaike Information Criterion. In the case of the Ng-Perron tests, the AR GLS detrended spectral estimation method is used with the lag length selected using the Modified Akaike Information Criterion [18] with the Perron-Qu modification [20].

1.3. Perron-Yabu testing procedure for structural changes in the trend function

It has been shown that the presence of structural changes can have considerable implications when investigating time-series properties by means of unit root tests [12]. This creates a circular problem given that most of the tests for structural breaks require to correctly identify whether the data generating process is stationary or integrated. Depending on whether the process is stationary or integrated the limit distributions of these tests are different and if the process is misidentified the tests will have poor properties.

The Perron-Yabu test [21] was designed explicitly to address the problem of testing for structural changes in the trend function of a univariate time series without any prior knowledge as to whether the noise component is stationary or contains an autoregressive unit root. The approach of Perron-Yabu builds on previous work of the

same authors who analyzed the problem of hypothesis testing on the slope coefficient of a linear trend model when no information about the nature, I(0) or I(1), of the noise component is available [22].

We discuss the case of an autoregressive noise component of order one (AR(1)). A more detailed presentation of this case and of other structural change models and extensions can be found in [21]. Consider the following data generating process:

$$y_t = x_t^{'}\psi + u_t$$
$$u_t = \alpha u_{t-1} + e_t$$

for $t=1,...,T$; e_t~i.i.d.$\left(0,\sigma^2\right)$, x_t is a $\left(r \times 1\right)$ vector of deterministic components, and ψ is a $\left(r \times 1\right)$ vector of unknown parameters which are model specific and described in the next paragraphs. The initial condition u_0 is assumed to be bounded in probability. The autoregressive coefficient is such that $\left|\alpha\right| \leq 1$ and therefore, both integrated and stationary errors are allowed.

The interest resides in testing the null hypothesis of $R\psi = \gamma$ where R is a $\left(q \times r\right)$ full rank matrix and γ is a $\left(q \times 1\right)$vector, where q is the number of restrictions. The restrictions here are used to test for the presence of a structural change in the trend function. For this purpose the Perron-Yabu test considers three models where a change of intercept and/or slope in the trend function occurs. In what follows, the break date is denoted $T_B = \left[\lambda_B T\right]$ for some $\lambda_B \in \left(0,1\right)$, where $\left[\cdot\right]$ denotes the largest integer that is less than or equal to the argument. $1\left(.\right)$ is the indicator function.

The model to test for a one-time change in the slope of the trend function is specified with $x_t = \left(1, t, DT_t^*\right)'$ and $\psi = \left(\mu_0, \beta_0, \beta_1\right)'$ where $DT_t^* = 1\left(t > T_B\right)\left(t - T_B\right)$ so that the

trend function is joined at the time of the break. The hypothesis of interest is $\beta_1 = 0$. The testing procedure is based on a Quasi Feasible Generalized Least Squares approach that uses a superefficient estimate of the sum of the autoregressive parameters α when α=1. The estimate of α is the OLS estimate obtained from an autoregression applied to detrended data and is truncated to take value 1 when the estimate is in a $T^{-\delta}$ neighborhood of 1. This makes the estimate "super-efficient" when α=1 and implies that inference on the slope parameter can be performed using the standard Normal or Chi-square distribution whether α=1 or |α|<1, when the break date is known. Theoretical arguments and simulation evidence show that δ=1/2 is the appropriate choice. When the break date is unknown, the limit distribution is nearly the same in the I(0) and I(1) cases when considering the Exp functional of the Wald test across all permissible dates for a specified equation. Hence, it is possible to have tests with nearly the same size in both cases. To improve the finite sample properties of the test, they also use the Roy-Fuller bias-corrected version of the OLS estimate of α [23].

The testing procedure suggested by the authors is:

1) For any given break date, detrend the data by Ordinary Least Squares (OLS) to obtain the residuals $\hat{u}_t$;

2) Estimate an AR(1) model for $\hat{u}_t$ yielding the estimate $\hat{\alpha}$;

3) Use $\hat{\alpha}$ to get the Roy-Fuller biased corrected estimates $\hat{\alpha}_M$;

4) Apply the truncation

$$\hat{\alpha}_{MS} = \begin{cases} \hat{\alpha}_M & if \quad |\hat{\alpha}_M - 1| > T^{-1/2} \\ 1 & if \quad |\hat{\alpha}_M - 1| \le T^{-1/2} \end{cases}$$

5) Apply a Generalized Least Squares (GLS) procedure with $\hat{\alpha}_{MS}$ to obtain the coefficients of the trend and the variance of the residuals and construct the standard Wald-statistic W_{FMS}.

6) Since the break date is assumed to be unknown, the 5 steps above must be repeated for all permissible break dates to construct the Exp functional of the Wald test denoted by

$$Exp - W_{FS} = \log\left[T^{-1}\sum_{\Lambda}\exp\left(\frac{1}{2}W_{FMS}(\lambda)\right)\right]$$

where $\Lambda = \{\lambda; \varepsilon \le \lambda \le 1-\varepsilon\}$ for some $\varepsilon > 0$. $\varepsilon = 0.15$, 0.10 and 0.05 are commonly used in the literature.

1.4. The Perron and Kim-Perron unit root tests

Perron [12] proposed an extension of the Augmented Dickey-Fuller (ADF) test [14]-[15] that allows for a one-time break in the trend function of a univariate time series. Three different model specifications were considered: the "crash" model that allows for an exogenous change in the level of the series; the "changing growth" model that permits an exogenous change in the rate of growth; and a third model that allows both changes. For this test, the break dates are treated as exogenous in the sense of intervention analysis [24], separating what can and cannot be explained by the noise in a time series. Our interest centers in the "changing growth" model, which can be briefly described as follows. The null hypothesis is:

$$y_t = \mu_1 + y_{t-1} + (\mu_2 - \mu_1)DU_t + e_t$$

where $DU_t = 1$ if $t > T_B$, 0 otherwise; T_B refers to the time of the break, and $A(L)e_t = B(L)v_t$, v_t ~i.i.d. $(0, \sigma^2)$, with *A(L)* and *B(L)* *p*th and *q*th order polynomials, respectively, in the lag operator. The innovation series $\{e_t\}$ are ARMA(*p,q*) type with possibly unknown *p, q* orders. The alternative hypothesis is:

$$y_t = \mu_1 + \beta_1 t + (\beta_2 - \beta_1) DT_t * + e_t$$

where $DT_t^* = t - T_B$ if $t > T_B$ and 0 otherwise. The "changing growth" model takes an "additive outlier" approach in which the change is assumed to occur rapidly and the regression strategy consists in first detrending the series according the following regression:

$$y_t = \mu + \beta_1 t + \gamma DT_t^* + \tilde{y}_t \qquad (4)$$

where $\gamma = (\beta_2 - \beta_1)$. Then an ADF regression is estimated on the residuals $\tilde{y}_t$ as follows:

$$\tilde{y}_t = \alpha \tilde{y}_{t-1} + \sum_{i=1}^{k} c_i \Delta \tilde{y}_{t-i} + e_t \qquad (5)$$

where the *k* lagged values of Δy_{t-i} are added as a parametric correction for autocorrelation. In the original Perron test [12] the break is assumed to occur at a known date. Later, this test was generalized for the case when the date of the break is unknown and he proposed determining the break point endogenously from the data

[25]. The break date was originally proposed to be estimated by 1) minimizing the t-statistic for testing $\alpha = 1$; 2) minimizing/maximizing the t-statistic of the parameter associated with γ in regression (4) or; 3) maximizing the absolute value of the t-statistic of γ in regression (4). The resulting unit root test is then the t-statistic for testing that α=1 in regression (5) estimated by OLS. The critical values of the limit distribution of the test have been tabulated [25].

A problem with most procedures for testing for unit roots in the presence of a one-time break that occurs at an unknown date is that the change in the trend function is allowed only under the alternative hypothesis of a stationary noise component [25]-[27]. Consequently, it is possible that a rejection occurs when the noise is I(1) and there is a large change in the slope of the trend function. A method that avoids this problem is that of Kim-Perron [28]. Their procedure is based on a pre-test for a change in the trend function, namely the Perron-Yabu test described above. If this pre-test rejects, the limit distribution of the unit root test is then the same as if the break date was known [12], [29]. This is very advantageous since when a break is present the test has much greater power. It was also shown in simulations to maintain good size in finite samples and that it offers improvements over other commonly used methods. The testing procedure under the additive outlier approach for the changing growth model consists in the following steps:

1. Obtain an estimate of the break date $\hat{T}_B = \hat{\lambda} T$ by minimizing the sum of squared residuals from regression (4). Then construct a window around that estimate defined by a lower bound T_l and an upper bound T_h. A window of 9

observations was used. Note that the results are not sensitive to this choice [28];

2. Create a new data set $\{y^n\}$ by removing the data from $T_l + 1$ to T_h, and shifting down the data after the window by $S(T) = y_{T_h} - y_{T_l}$, hence,

$$y^n = \begin{cases} y_t & if \quad t \le T_l \\ y_{t+t_h-t_l} - S(T) & if \quad t > T_l \end{cases}$$

3. Perform the unit root test using the break date T_l and compute the *t*-test statistic for testing $\tilde{\alpha} = 1$, denoted by $t_\alpha\left(\hat{\lambda}_{tr}^{AO}\right)$, from the following OLS regression:

$$\tilde{y}_t^n = \tilde{\alpha}\tilde{y}_{t-1}^n + \sum_{i=1}^{k} \tilde{c}_i \, \Delta\tilde{y}_{t-i}^n + \tilde{e}_t \qquad (6)$$

where $\hat{\lambda}_{tr}^{AO} = T_l / T_r$, $T_r = T - (T_h - T_l)$ and $\tilde{y}_t^n$ is the detrended value of y^n.

The number of lags in (5) and (6) was chosen using the Schwarz Information Criterion (BIC) but the results are in general robust to alternative methods for choosing the lag length such as the Akaike Information Criterion (AIC) or the Hannan-Quinn criterion (HQ). In all cases, no evidence of remaining autocorrelation was found based on Ljung-Box tests applied to the residuals.

1.5. Bierens nonparametric nonlinear co-trending test

Nonlinear co-trending is special case of the more general "common features" concept [30]. The advantage of the test proposed by Bierens is that the nonlinear trend does not have to be parameterized [31]. The nonlinear trend stationarity model can be expressed as follows:

$$z_t = g(t) + u_t$$

with

$$g(t) = \beta_0 + \beta_1 t + f(t)$$

where z_t is a k-variate time series, u_t is a k-variate zero-mean stationary process and $f(t)$ is deterministic k-variate general nonlinear trend function that allows, in particular, structural changes. Nonlinear co-trending occurs when there exists a non-zero vector θ such that $\theta' f(t) = 0$. Hence, the null hypothesis of this test is that the multivariate time series z_t is nonlinear co-trended, implying that there is one or more linear combinations of the time series that are stationary around a constant or a linear trend. Note that this test is a cointegration test in the case when it is applied to series that contain unit roots.

The nonparametric test for nonlinear co-trending is based on the generalized eigenvalues of the matrices M_1 and M_2 defined by:

$$M_1 = \frac{1}{n}\sum_{t=1}^{n} \hat{F}\left(\frac{t}{n}\right)\hat{F}\left(\frac{t}{n}\right)'$$

where

$$\hat{F}(x) = \frac{1}{n}\sum_{t=1}^{[nx]} \left(z_t - \hat{\beta}_0 - \hat{\beta}_1 t\right)$$

if $x \in [n^{-1}, 1]$, $F(x) = 0$ if $x \in [0, n^{-1}]$ with $\hat{\beta}_0$ and $\hat{\beta}_1$ being the estimates of the vectors of intercepts and slope parameters in a regression of z_t on a constant and a time trend; and

$$M_2 = \left(\frac{1}{n}\right)\sum_{j=0}^{m-1}\left[\left(\frac{1}{m}\right)\sum_{j=0}^{m-1}\left(z_{t-j} - \hat{\beta}_0 - \hat{\beta}_1(t-j)\right)\right]\left[\left(\frac{1}{m}\right)\sum_{j=0}^{m-1}\left(z_{t-j} - \hat{\beta}_0 - \hat{\beta}_1(t-j)\right)\right]'$$

where $m = n^{\alpha}$ with n equal to the number of observations and $\alpha = 0.5$ [31]. Solving $\left|M_1 - \lambda M_2\right| = 0$ and denoting the solution $\hat{\lambda}_r$, the test statistic is $n^{1-\alpha}\hat{\lambda}_r$. The null hypothesis is that there are *r* co-trending vectors against the alternative of *r-1* co-trending vectors. This test has a non-standard distribution and the critical values have been tabulated [31]. The existence of *r* co-trending vectors in *r+1* series indicates the presence of *r* linear combinations of the series that are stationary around a linear trend and that these series share a single common nonlinear deterministic trend. Such a result indicate a strong secular co-movement in the *r+1* series.

1.6 Robustness of the unit root test results

In this subsection the robustness of the results about the unit root tests presented in Table 1 of the main text is explored by comparing them to those that can be obtained using the Perron test described above [25]. For this purpose, two of the different methods for selecting the break date for this test were applied. Although both methods are equivalent to choosing the break date by minimizing the sum of squared residuals, the test and the appropriate critical values in each case are different, as explained below.

The first method consists in maximizing the value of the of the t-statistic of coefficient γ in regression (4) to obtain the test statistic $t^*_{\alpha,\gamma}$ from regression (5). This method allows to impose a mild prior restriction of a one-sided change ($\gamma > 0$), limiting the analysis to the case of interest of the present paper (i.e., when there is an

increase in the rate of growth). This restriction increases the power of the test when there is indeed a non-zero change in the slope [25]. The existence of breaks in the slope of the trend function of temperature and radiative forcing series was previously established using the Perron-Yabu test [21], and consequently this is the relevant test statistic for investigating their time-series properties. The second method does not impose any restriction on γ and consists in maximizing the absolute value of the t-statistic of the coefficient γ to obtain the test statistic $t^*_{\alpha,|\gamma|}$. The results obtained following this procedure are more conservative since no information about the sign of the change is included and the corresponding critical values are larger in absolute value. Note that both tests yield exactly the same values. The difference between the two relates to the assessment of the significance of a given value for the statistic. If one imposes a prior that the change is positive, the critical values are smaller (in absolute value) and, hence, the tests is more powerful.

Table A1 provides strong evidence on the robustness of the results shown in Table 1 of the main text. Whether the restriction of a one-sided test is imposed or not, results unambiguously indicate that both the temperature simulations and radiative forcing series are better represented as trend stationary processes with a one-time break in the slope of their trend function. For all temperature simulations both $t^*_{\alpha,\gamma}$ and $t^*_{\alpha,|\gamma|}$ are significant at the 1% level, with the exception of GFDL_CM2.1_3, for which they are significant at the 2.5% and 5% levels, respectively. For the radiative forcing series the $t^*_{\alpha,\gamma}$ is significant at the 1%, 2.5% and 5% levels for SOLAR, TRF and WM_GHG, respectively. The results are broadly similar even when using the more conservative critical values corresponding to $t^*_{\alpha,|\gamma|}$. In this case, the test statistic is significant at the

1%, 2.5% and 5% levels for SOLAR, TRF and WM_GHG, respectively. As such, the conclusion obtained by applying the Kim-Perron test [28] in that both temperature simulations and radiative forcing series can be better described as trend stationary processes with a change in their rates of growth is strongly supported by the Perron test [25], whether or not a prior restriction on the sign of the change is imposed.

Table A1. Perron test for a unit root with a one-time break in the trend function. The regression model for the unit root tests is defined in equations (4) and (5). The values of the estimated parameters are reported in Table 1 of the main text. a, b, c, d denotes statistical significance at the 1%, 2.5%, 5% and 10% respectively. The critical values are from [25] Table 1, panels (h) and (i).

Series	$t^*_{\alpha,\gamma}$, $t^*_{\alpha,\lvert\gamma\rvert}$	Significance level imposing the prior of a positive slope change	Significance level without a prior imposed
Globe	-6.6978	a	a
ECHAM5_1	-10.5027	a	a
ECHAM5_2	-6.7142	a	a
ECHAM5_3	-11.1100	a	a
ECHAM5_4	-6.2068	a	a
BCCR	-6.7843	a	a
CCCMA	-9.0049	a	a
GFDL_CM2.1_1	-5.0001	a	a
GFDL_CM2.1_3	-4.5615	b	c
GFDL_CM2.0_1	-5.4219	a	a
HADLEY_CM3_1	-5.7231	a	a
HADLEY_CM3_2	-8.0815	a	a
GISS_AOM_1	-8.2308	a	a
GISS_AOM_2	-6.3705	a	a
IPSL	-10.1338	a	a
TRF	-4.5822	b	b
WM_GHG	-4.2380	c	d
SOLAR	-9.3831	a	a

References

1. Maddala GS, Kim IM (1998) Unit Roots, Cointegration and Structural Change. Cambridge, UK: Cambridge University Press. 524 p.

2. Hatanaka M (1996) Time-Series-Based Econometrics. Unit Roots and Cointegration. Oxford, UK: Oxford University Press. 308 p.

3. Gay C, Estrada F, Sanchez A (2009) Global and hemispheric temperature revisited. Clim Change 94: 333–349.

4. Gay C, Estrada F, Conde C (2007) Some implications of time series analysis for describing climatologic conditions and for forecasting. An illustrative case: Veracruz, Mexico. Atmosfera 20(2): 147-170.

5. Peixoto JP, Oort AH (1992) Physics of climate. New York, USA: American Institute of Physics. 520 p.

6. Engle RF, Granger CWJ (1987) Co-integration and error correction: Representation, estimation and testing. Econometrica 55: 251-276.

7. Stock JH, Watson MW (1988) Variable trends in economic time series. J Econ Perspect 2(3): 1097-1107.

8. Gonzalo J, Lee TH (1998) Pitfalls in testing for long run relationships. J Econometrics 86: 129-154.

9. Elliott G (1998) On the robustness of cointegration methods when regressors almost have unit roots. Econometrica 66: 149-158.

10. Leybourne SJ, Newbold P (2003) Spurious rejections by cointegration tests induced by structural breaks. App Econ 35: 1117-1121.

11. Enders W (2003) Applied Econometric Time Series. New York, USA: Wiley. 544 p.

12. Perron P (1989) The great crash, the oil price shock, and the unit root hypothesis. Econometrica 57: 1361–1401.

13. Perron P, Wada T (2009) Let's take a break: Trends and cycles in US real GDP. J Monet Econ 56: 749-765.

14. Dickey DA, Fuller WA (1979) Distribution of the Estimators for Autoregressive Time Series with a Unit Root. J Am Statist Assoc 74: 427–431.

15. Said E, Dickey DA (1984) Testing for unit roots in autoregressive moving average models of unknown order. Biometrika 71: 599–607.

16. Kwiatkowski D, Phillips PCB, Schmidt P, Shin Y (1992) Testing the null hypothesis of stationarity against the alternative of a unit root. J Econom 54: 159-178.

17. Elliott G, Rothenberg TJ, Stock JH (1996) Efficient tests for an autoregressive unit root. Econometrica 64: 813–836.

18. Ng S, Perron P (2001) Lag Length Selection and the Construction of Unit Root Tests with Good Size and Power. Econometrica 69: 1519-1554.

19. Newey WK, West KD (1994) Automatic Lag Selection in Covariance Estimation. Rev Econ Stud 61: 631-654.

20. Perron P, Qu Z (2007) A simple modification to improve the finite sample properties of Ng and Perron's unit root tests. Econ Letters 94: 12-19.

21. Perron P, Yabu T (2009) Testing for shifts in trend with an integrated or stationary noise component. JBES 27: 369-396.

22. Perron P, Yabu T (2009) Estimating deterministic trends with an integrated of stationary noise component. J Econom 151: 56-69.

23. Roy A, Fuller WA (2001) Estimation for autoregressive processes with a root near one. JBES 19: 482-493.

24. Box GEP, Tiao GC (1975) Intervention Analysis with Applications to Economic and Environmental Problems. J Amer Statistical Assoc 70: 70-79.

25. Perron P (1997) Further evidence on breaking trend functions in macroeconomic variables. J Econom 80: 355-385.

26. Zivot E, Andrews D (1992) Further evidence on the great crash, the oil price shock, and the unit root hypothesis. JBES 10: 251–270.

27. Vogelsang TJ, Perron P (1998) Additional tests for a unit root allowing the possibility of breaks in the trend function. Int Econ Rev 39: 1073-1100.

28. Kim D, Perron P (2009) Unit root tests allowing for a break in the trend function under both the null and the alternative hypotheses. J Econometrics 148: 1-13.

29. Perron P, Vogelsang T (1993) Erratum: The great crash, the oil price shock and the unit root hypothesis. Econometrica 61: 248-249.

30. Engle RF, Kozicki S (1993) Testing for common features. JBES 11: 369-395.

31. Bierens HJ (2000) Nonparametric nonlinear cotrending analysis, with an application to interest and inflation in the United States. JBES 18: 323-337.
